# Chimeric Mice with Humanized Livers: A Unique Tool for *in Vivo* and *in Vitro* Enzyme Induction Studies

**DOI:** 10.3390/ijms15010058

**Published:** 2013-12-20

**Authors:** Masakazu Kakuni, Chihiro Yamasaki, Asato Tachibana, Yasumi Yoshizane, Yuji Ishida, Chise Tateno

**Affiliations:** 1 PhoenixBio Co., Ltd., 3-4-1, Kagamiyama, Higashihiroshima, Hiroshima 739-0046, Japan; E-Mails: masakazu.kakuni@phoenixbio.co.jp (M.K.); chihiro.yamasaki@phoenixbio.co.jp (C.Y.); atachibana@phoenixbio.co.jp (A.T.); yyoshizane@phoenixbio.co.jp (Y.Y.); yuji.ishida@phoenibio.co.jp (Y.I.); 2 Liver Research Project Center, Hiroshima University, 1-2-3 Kasumi, Minami, Hiroshima, Hiroshima 734-8551, Japan

**Keywords:** liver, P450 induction, humanized animal model, rifampicin, 3-methylcholanthrene

## Abstract

We performed *in vivo* and *in vitro* studies to determine the induction of human cytochrome P450 (CYP) using chimeric mice with humanized liver (PXB-mice^®^) and human hepatocytes isolated from the PXB-mice (PXB-cells), which were derived from the same donor. For the *in vivo* study, PXB-mice were injected with 3-methylcholanthrene (3-MC, 2 or 20 mg/kg) or rifampicin (0.1 or 10 mg/kg) for four days. For the *in vitro* study, PXB-cells were incubated with 3-MC (10, 50, or 250 ng/mL) or with rifampicin (5 or 25 μg/mL). The *CYP1A1* and *1A2*, and *CYP3A4* mRNA expression levels increased significantly in the PXB-mouse livers with 20 mg/kg of 3-MC (*C*_max_, 12.2 ng/mL), and 10 mg/kg rifampicin (*C*_max_, 6.9 μg/mL), respectively. The *CYP1A1* mRNA expression level increased significantly in PXB-cells with 250 ng/mL of 3-MC, indicating lower sensitivity than *in vivo*. The *CYP1A2* and *CYP3A4* mRNA expression levels increased significantly with 50 ng/mL of 3-MC, and 5 μg/mL of rifampicin, respectively, which indicated that the sensitivities were similar between *in vivo* and *in vitro* studies. In conclusion, PXB-mice and PXB-cells provide a robust model as an intermediate between *in vivo* and *in vitro* human metabolic enzyme induction studies.

## 1. Introduction

Metabolic enzyme induction is a side effect of some drugs, and it can cause important problems in drug metabolism and toxicity, such as a reduction in a drug’s effect and an increase in reactive metabolites. It is, thus, necessary to evaluate the induction potential of drugs in humans during preclinical drug development. However, such predictions are difficult to test because there are species differences between humans and laboratory animals in metabolic enzyme inducibility. There is also limited availability of donated human liver samples.

Previously, chimeric mice with humanized livers were generated by transplanting cryopreserved human hepatocytes into the spleen of urokinase-type plasminogen activator (uPA)/severe combined immunodeficient (SCID) mice [1,2]. In the liver of the chimeric mouse (PXB-mouse^®^) we developed, mouse hepatocytes are largely repopulated with the transplanted human hepatocytes, which have been demonstrated to express human cytochrome P450 (CYP) enzymes [3], phase II enzymes [4], and transporters [5], and have the potential for CYP enzyme induction with inducers [2]. Recently, other human liver chimeric mice were generated using *Fah*^−/−^/*Rag2*^−/−^/*Il2rg*^−/−^ mice and TK-NOG mice and humanized livers from these mice expressed human CYP mRNA at similar levels as human hepatocytes [6,7]. As treatment of the mice with inducers results in *in vivo* enzyme induction in the humanized hepatocytes, the animals chimeric mice enable the evaluation of enzyme inducing effects would be useful in predicting enzyme induction in humans.

*In vitro* enzyme induction studies are routinely conducted during drug development at pharmaceutical companies, and very large numbers of human hepatocytes are used in such studies to predict the potential for enzyme induction. Many of the human hepatocytes used in such *in vitro* enzyme induction studies are supplied fresh, due to advantages in terms of cell function, primarily plating efficiency in dishes, as compared with cryopreserved human hepatocytes. However, it is difficult to obtain fresh human hepatocytes for *in vitro* studies, including enzyme induction studies, due to their limited availability. Additionally, preparing fresh human hepatocytes ahead of time and performing reproducible studies using the same donor cells are not possible.

In contrast, our chimeric mice possess live human hepatocytes in the liver and fresh human hepatocytes from the chimeric mouse (PXB-cells) are thus considered to be a suitable model to be used in place of fresh human hepatocytes for *in vitro* studies. The availability of cryopreserved human hepatocytes isolated from the chimeric mice has been established in evaluating the induction of hCYP1A2 and hCYP3A4 in previous studies [8,9] and a recent study demonstrated repeated and on-demand availability of fresh chimeric human hepatocytes derived from the same donor using these chimeric mice [10]. However, there have been no studies in which induction abilities of human hepatocytes were directly compared between *in vivo* and *in vitro* conditions.

In the present study, we investigated enzyme induction *in vivo* in the intact chimeric mice, and *in vitro* using fresh chimeric human hepatocytes derived from the same donor. Our result demonstrates that the unique *in vivo*/*in vitro* human hepatocyte model provides robust information to prove the molecular mechanism of differentiation in three-dimensional (3-D) morphology, and bridge between *in vivo* and *in vitro* pharmacological studies.

## 2. Results and Discussion

### 2.1. Results

#### 2.1.1. Induction of CYPs in 3-Methylcholanthrene- (3-MC) and Rifampicin-Administered Chimeric Mouse Liver

**Figure 1.**
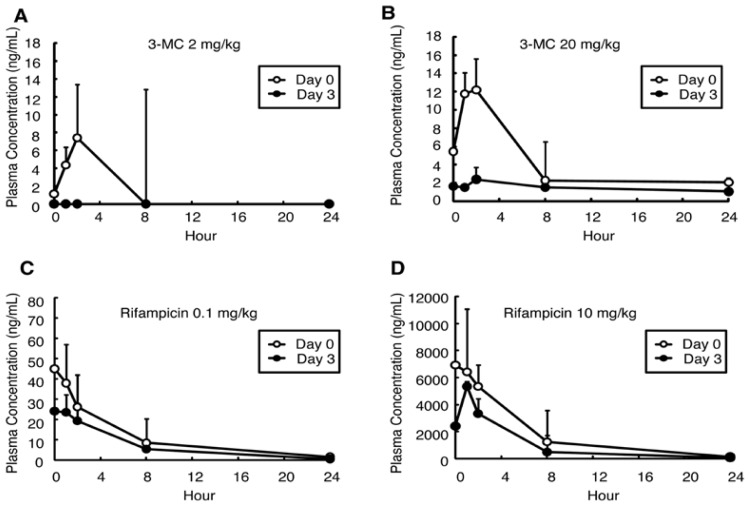
Plasma concentrations of 3-MC or Refampicin in the *in vivo* study. Plasma concentrations of 3-MC (**A**,**B**) and rifampicin (**C**,**D**) were measured on the first (Day 0) and the last (Day 3) day of administration. Open circles indicate data on Day 0. Closed circles indicate data on Day 3. Data are mean ± S.D.

3-Methylcholanthrene (3-MC) was administered intraperitoneally to groups consisting of three chimeric mice (PXB-mice^®^), each at a dose of 2 or 20 mg/kg daily for four days. In both the 2 and 20 mg/kg 3-MC-administered groups, the *AUCs* of 3-MC decreased at Day 3 as compared with Day 0 (Figure 1A, Table 1). In the 20 mg/kg group, the *AUC* decreased from 94.4 ng/mL·h on Day 0 to 35.4 ng/mL·h on Day 3; the difference was significant (0.4-fold; *p* < 0.01; Figure 1B, Table 1). The reductions in *AUC* were accompanied by decreases in *C*_max_ (Table 1). Rifampicin was also administered intraperitoneally to groups of three mice, at dose levels of 0.1 or 10 mg/kg daily for four days. After the four days of administration, the *AUCs* of rifampicin decreased in both 0.1 (0.7-fold, Figure 1C, Table 1) and 10 mg/kg-administered groups (0.6-fold, Figure 1D, Table 1), but the difference was not significant. The decreases in *C*_max_ were confirmed in both groups.

mRNA expression levels were determined by real-time quantitative RT-PCR (qRT-PCR) using liver samples collected at 24 h after the last administration on Day 4. The mRNA expression levels of h*CYP1A1*, h*CYP1A2*, and h*CYP3A4* were normalized to the expression levels of human *glyceraldehyde 3-phosphate dehydrogenase* (h*GAPDH*) (Table 2). Although the chimeric mouse livers contained mouse hepatocytes (<30%), real-time qRT-PCR determined the gene expression levels of only human hepatocytes but not mouse hepatocytes because the primers were human-specific. The mRNA expression levels of h*CYP1A1* and h*CYP1A2* increased in the 20 mg/kg 3-MC-administered group, and the change in h*CYP1A1* was significant as compared with the control group (7.3-fold, *p* < 0.05, Table 2). The mRNA expression levels of h*CYP3A4* increased significantly in the 10 mg/kg rifampicin-administered group (5.1-fold, *p* < 0.05, Table 2).

**Figure 2.**
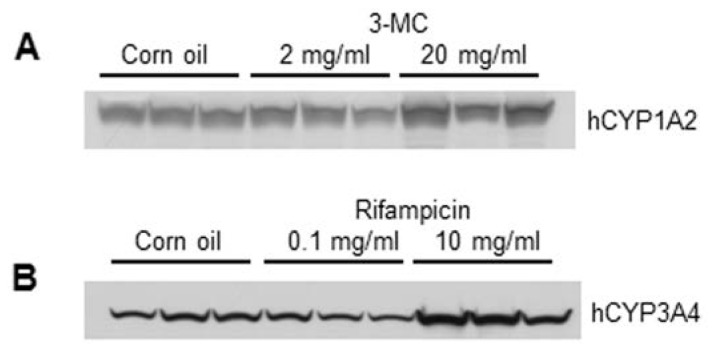
Protein expression levels in the *in vivo* study. Changes in hCYP1A2 expression induced by four days of 3-MC administration (**A**) and in hCYP3A4 expression induced by four days of rifampicin administration (**B**) are shown.

Protein expression levels of human CYPs were determined by Western blotting using microsomal fractions isolated from the mouse liver. The protein expression level of hCYP1A2 increased significantly in the 20 mg/kg 3-MC group (1.5-fold, *p* < 0.05, Figure 2, Table 3), and the protein expression level of hCYP3A4 in the 10 mg/kg rifampicin group increased significantly (2.2-fold; *p* < 0.05) as compared with the control group (Figure 2, Table 3).

#### 2.1.2. Regional Distribution of hCYP1A2 and 3A4 Expression with 3-MC and Rifampicin Administration

**Figure 3.**
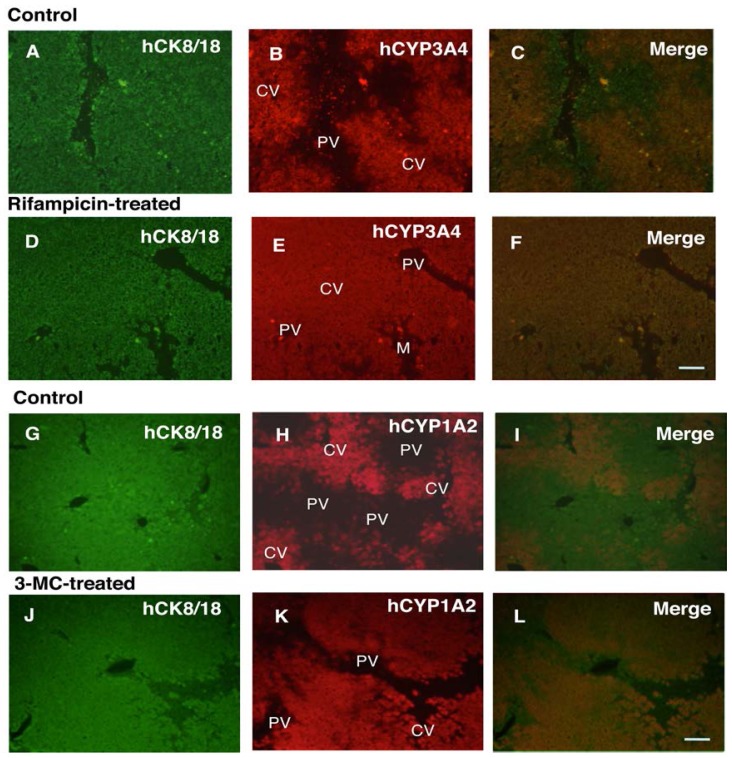
Double immunohistochemistry for human cytokeratin 8/18 (CK8/18) and hCYP1A2, and CK8/18 and hCYP3A4 in chimeric mouse livers treated with inducers (3-MC, rifampicin). hCK8/18 (**A**,**D**,**G**,**J**) was visualized with FITC (green color), and hCYP3A4 (**B**,**E**) and hCYP1A2 (**H**,**K**) were visualized with Texas Red (red color). The overlays of hCK8/18 and hCYP3A4 or hCK8/18 and hCYP1A2 are shown in (**C**,**F**,**I**,**L**). (**A**–**C**,**G**–**I**) was derived from non-treated animals. (**D**–**F**) was derived from rifampicin-treated animals and (**J**–**L**) was derived from 3-MC-treated animals. H, human hepatocyte-region; M, mouse hepatocyte-region. CV, central vein; PV, portal vein. Bar, 100 μm. Magnifications of (**A**–**E**) and (**F**–**K**) are same as those of (**F**) and (**L**), respectively.

The localization of hCYP1A2 and 3A4 was immunohistochemically determined using cryosections of livers from untreated and 3-MC- or rifampicin-administered chimeric mice. Both hCYP1A2 and 3A4 were located in the centrilobular, but not the periportal area in control chimeric mouse livers (Figure 3A–C,G–I). After four days of 3-MC or rifampicin administration, the expression of these CYPs expanded to the periportal area (Figure 3D–F,J–L).

#### 2.1.3. *In Vitro* Induction of CYPs in 3-MC- or Rifampicin-Treated Human Hepatocytes

**Figure 4.**
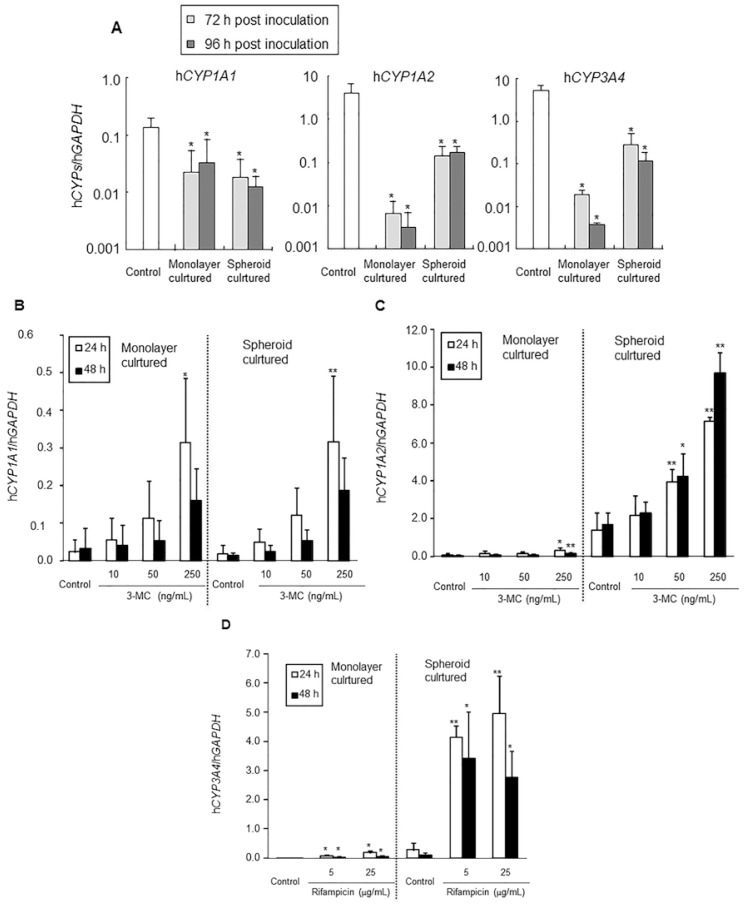
mRNA expression levels in the *in vitro* study. The baseline mRNA expression levels of h*CYP1A1*, h*CYP1A2* and h*CYP3A4* in monolayer culture and spheroid culture of human hepatocytes after inoculation are shown in (**A**); Changes in the value of h*CYP1A1*/h*GAPDH*, h*CYP1A2*/h*GAPDH*, and h*CYP3A4*/h*GAPDH* by inducers are shown in (**B**,**C**) and (**D**), respectively. Data are mean ± S.D. * *p* < 0.05; ** *p* < 0.01.

Fresh human hepatocytes (PXB-cells) were isolated from chimeric mice by a two-step collagenase perfusion method, and prepared as monolayer and spheroid cultures. In the present study, the chimeric mouse hepatocytes contained <10% mouse hepatocytes. Similar to the *in vivo* study, real-time qRT-PCR determined the gene expression levels of only human hepatocytes but not mouse hepatocytes because the primers were human specific. The baseline mRNA expression levels of hCYP1A1, hCYP1A2, and hCYP3A4 in monolayer and spheroid cultures were confirmed at 72 and 96 h post inoculation as compared with the control (immediately after isolation). For hCYP1A1, mRNA expression levels were maintained at more than 10% of the *in vivo* control in both monolayer and spheroid cultures at 72 and 96 h (Figure 4A, *p* < 0.05). For hCYP1A2 and hCYP3A4, mRNA expression levels decreased to less than 1% of the control in the monolayer culture, while in the spheroid culture, expression was maintained at around 10% of the control at 72 and 96 h (Figure 4A, *p* < 0.05), suggesting the 3-D culture maintained human hepatocytes in more differentiated phase than monolayer culture.

Isolated human hepatocytes were incubated with 3-MC at 10, 50, or 250 ng/mL, or with rifampicin at 5 or 25 μg/mL. The incubation times were set at 24 (72 h after inoculation) and 48 h (96 h after inoculation). To evaluate enzyme induction, mRNA expression levels of h*CYP1A1*, h*CYP1A*2, and h*CYP3A4* were determined by real-time qRT-PCR and normalized to the expression level of h*GAPDH*. The normalized mRNA expression levels of h*CYP1A1* increased in a concentration-dependent manner in both monolayer and spheroid cultures, and the induction ratios were quite similar between them. Significant differences were observed in monolayer (14.3-fold, *p* < 0.05) and spheroid cultures (17.3-fold, *p* < 0.01) treated with 250 ng/mL 3-MC at 24 h (Table 4, Figure 4B). Although the expression levels of *CYP1A2* were less than 0.1-fold lower in monolayer culture than spheroid culture, the mRNA expression levels of h*CYP1A2* also increased in a concentration-dependent manner in both monolayer culture and spheroid culture, and the induction ratios were similar between them. Significant differences were observed in monolayer cultures treated with 250 ng/mL 3-MC at both 24 (4.9-fold, *p* < 0.05) and 48 h (4.9-fold, *p* < 0.01), and in monolayer cultures treated with 50 ng/mL 3-MC at both 24 (2.8-fold, *p* < 0.01) and 48 h (2.5-fold, *p* < 0.05) and with 250 ng/mL 3-MC at both 24 (5.1-fold, *p* < 0.01) and 48 h (5.8-fold, *p* < 0.01; Table 4, Figure 4C).

Although the mRNA expression levels of *CYP3A4* were less than 0.1-fold lower in monolayer culture than spheroid culture, similar to *CYP1A2* expression, concentration-dependent increases were confirmed at 24 and 48 h in both monolayer and spheroid cultures. All of these increases were significant compared with the control, and the induction ratio was higher in spheroid culture than monolayer culture (Table 4, Figure 4D).

### 2.2. Discussion

The present study was conducted to demonstrate the potential usefulness of chimeric mice with humanized livers in both *in vivo* and *in vitro* enzyme induction studies. In the *in vivo* study, a significant decrease in *AUC* of 3-MC was induced by four days 3-MC administration (20 mg/kg), which was associated with a significant increase in h*CYP1A1* mRNA expression and hCYP1A2 protein expression. *AUC* decreases were observed at 2 mg/kg in the 3-MC group (but not significant) although the expression levels of mRNA (h*CYP1A1* and h*CYP1A2*) and protein (hCYP1A2) did not increase significantly after dosing. These results show that although 3-MC is known to be metabolized by CYP1A [11], we expected that up-regulation of mRNA or protein of, not only CYP1A1 and 1A2, but also other metabolic enzymes or transporters might contribute to the apparent auto-induction by 3-MC. There was no significant difference in *AUC* levels between the control and rifampicin-treated groups; however, the *AUC* decreased by about one-half in the 10 mg/kg rifampicin group, and this change was associated with a significant increase in h*CYP3A4* mRNA and protein expression levels. Although the exact mechanism is not known, chronic dosing of rifampicin induces its own metabolism [12]. In addition, the *AUCs* in 10 mg/kg of rifampicin-administered group were similar to the *AUC* at steady state (*AUCss*) of human treated with a therapeutic dose of rifampicin (22,400 to 35,300 ng·h/mL [13]). Rifampicin is a potent and selective activator of the human nuclear pregnane X receptor (PXR). It has been reported that there are significant differences in ligand recognition by PXR between rodents and humans [14]. Although the chimeric mice retain less than 30% mouse hepatocytes, decreases in *AUC* might have occurred by PXR-related induction in human hepatocytes but not in mouse hepatocytes. Recently we reported that CYP3A4 and CYP2C subfamilies were induced by treatment of the PXB mice with 50 mg/kg rifampicin for four days. However treatment with 10 mg/kg decreased only the *AUC* of the CYP3A4 substrate, and did not affect the *AUC* of CYP2C substrates [15]. These data suggest that induction of CYP3A4 might contribute to the *AUC* decrease of rifampcin. On the other hand, as 3-MC induces mouse Cyp1a1 and hCYP1A [11], the Cyp1a1 induction in less than 30% mouse hepatocytes in the chimeric liver might contribute to 3-MC auto-induction.

The protein induction of hCYP1A2 and hCYP3A4 in the chimeric mice was supported by the immunohistochemistry results. In the *in vivo* situation, there is morphological, biochemical, molecular, and functional heterogeneity between periportal and pericentral hepatocytes [16–20]. CYP3A and CYP1A2 are expressed in pericentral, but not in periportal, hepatocytes in rodent and human liver lobules [19,20]. It has been reported that administering phenobarbital to rats induces the expression of CYP3a1 in periportal hepatocytes [20]. In the present study, we demonstrated that *in vivo* treatment of human hepatocytes with rifampicin and 3-MC induced hCYP3A4 and hCYP1A2 expression in periportal hepatocytes of the chimeric mice, resulting in uniform expression of these enzymes throughout the liver lobules. These data are consistent with our previous study showing induction of CYP3A4 in rifampicin-treated chimeric mice [21]. These results demonstrated that chimeric mice with humanized liver are appropriate for evaluating enzyme induction in human hepatocytes, based on *in vivo* exposure levels of the inducer and tissue levels.

Recently, we demonstrated that these chimeric mice may be useful for supplying fresh human hepatocytes on demand, thus, promising high and stable phase I enzyme and glucuronidation activities [10]. In the present *in vitro* study, we also demonstrated the feasibility of conducting 24- or 48-h enzyme induction studies in both monolayer and spheroid cultures using human hepatocytes freshly isolated from the chimeric mice. Given the scarcity of fresh human hepatocytes for conducting *in vitro* studies, fresh human hepatocytes isolated from chimeric mice may be a viable alternative. Additionally, it is possible to reproducibly conduct *in vitro* enzyme induction studies with fresh human hepatocytes derived from the same donor and to compare results of *in vitro* and *in vivo* studies using cells from the same donor.

For *in vitro* CYP induction study, hepatocytes are typically cultured in monolayer conditions. However, because CYP mRNAs, proteins, and activities decline immediately during monolayer culture, induction levels have been estimated at much lower levels (less than 1%) than *in vivo* levels [8]. In the present study, we compared *CYP1A1*, *1A2*, and *3A4* mRNA expression levels between hepatocytes just after isolation and monolayer- or spheroid-cultured hepatocytes at 72 and 96 h. *CYP1A2* and *3A4* mRNA expression levels in hepatocytes in both culture conditions decreased; however, they were maintained at more than 10-fold higher levels in spheroid cultures than in monolayer cultures, although *CYP1A1* mRNA expression levels were similar between hepatocytes in the two conditions. Based on these results, hepatocytes in the spheroid culture apparently maintain their differentiated state better than those in monolayer culture. Thus, we consider the spheroid-cultured hepatocytes to be hepatocytes with differentiation characteristics between those *in vivo* and in monolayer culture. *In vitro* CYP induction studies were performed in monolayer and spheroid cultures. Induction responsiveness and induction ratios of *CYP1A1* were similar between the two conditions. On the other hand, sensitivities of *CYP1A2* were higher in spheroid culture (50 ng/mL) than monolayer culture (250 ng/mL), however, the induction ratios were quite similar between them at the highest dose (3-MC 250 ng/mL). The sensitivity of *CYP3A4* was also similar (5 μg/mL), but induction ratios were higher in spheroid than monolayer cultures. From these results, sensitivities and induction ratios were similar or somewhat higher in spheroid than monolayer cultures.

Information from these *in vivo* and *in vitro* enzyme induction studies using human hepatocytes from the same donor may be useful in advancing predictions of enzyme induction in humans. For example, in the present study, 12.2 ng/mL of 3-MC in the *in vivo* study (20 mg/kg, Day 0) was sufficient to induce a significant decrease in *AUC* of 3-MC and a significant increase in h*CYP1A1* mRNA expression and hCYP1A2 protein expression. On the other hand, the *in vitro* study revealed that 250 ng/mL of 3-MC was needed to induce a significant increase in h*CYP1A1* mRNA in both monolayer and spheroid cultures, and 250 and 50 ng/mL 3-MC was needed to induce a significant increase of h*CYP1A2* mRNA in the monolayer and spheroid cultures, respectively, as shown above. The sensitivity to 3-MC was higher in spheroid than monolayer cultures for both hCYP1A1 and 1A2. The sensitivity of *CYP1A1* and *1A2* mRNA expression levels to 3-MC was in the following sequence: *in vivo* > spheroid ≥ monolayer. However, the sensitivity of CYP3A4 for rifampicin was similar between *in vivo* and *in vitro*.

## 3. Experimental Section

### 3.1. Materials

3-MC and rifampicin were purchased from Sigma-Aldrich (St. Louis, MO, USA). All other chemicals and vehicle were of analytical grade or the highest commercially available quality.

### 3.2. Generation of Chimeric Mice with Humanized Livers

The present study was approved by the Ethics Committees of PhoenixBio. To generate chimeric mice with humanized livers, cryopreserved human hepatocytes, which had been donated with informed consent, were purchased from BD Bioscience, Woburn, MA, USA (10YF, 10-year-old female Caucasian). Chimeric mice with humanized livers (PXB-mice^®^) were generated using a previously reported method [2]. Briefly, human hepatocytes were transplanted into the spleen of uPA/SCID mice at two to four weeks of age. From three weeks after the transplantation, 2 μL of blood was collected from the mice once per week and the concentration of human albumin (hAlb) in the blood was determined based on latex agglutination immunonephelometry to estimate the replacement index (RI, repopulation ratio of human hepatocytes in the host mouse liver). The correlation between hAlb concentration and the RI was established in a previous report [2]. In the present study 152 mice were transplanted with human hepatocytes and 74 mice (48.7%) showed >6 mg/mL hAlb (RI > 70%). The specifications of the chimeric mice used for the *in vivo* study were as follows: male, 12–14 weeks old, 6.3–10.9 mg/mL hAlb in the blood (RI > 70%), and 14.2–22.8 g body weight (Table 5). For the *in vitro* study, chimeric mice with 6.2–14.8 mg/mL hAlb in the blood (RI > 70%) were used to isolate human hepatocytes (Table 6).

### 3.3. *In Vivo* CYP Induction Study

3-MC and rifampicin were suspended in corn oil. Three chimeric mice per group were intraperitoneally administered corn oil, 2, or 20 mg/kg 3-MC, or 0.1 or 10 mg/kg rifampicin daily for four days (10 mL/kg). Blood was collected from the treated mice at 0.5, 1, 2, 8, and 24 h post first (Day 0) and last dosing (Day 3), and the plasma was used to determine concentrations of 3-MC and rifampicin. After the last blood sampling, these mice were euthanized under anesthesia, and the livers were harvested for the isolation of total RNA and microsomes.

### 3.4. Measurement of 3-MC and Rifampicin Concentrations in Mouse Plasma

To compare the effects of inducers between *in vivo* and *in vitro* studies, the plasma concentrations of 3-MC and rifampicin were measured by Sekisui Medical Co., Ltd. (Tokyo, Japan). A plasma sample (2 μL) was mixed well with 2 μL of acetonitrile and 60 μL of internal control solution, and centrifuged (22,000 ×*g*, 4 °C, 10 min). The supernatant was then applied to liquid chromatography-tandem mass spectrometry (LC-MS/MS; MDS SCIEX; Applied Biosystems, Foster City, CA, USA). Area under the curves (*AUCs*) were calculated based on the concentrations of 3-MC and rifampicin.

### 3.5. Immunohistochemistry

To evaluate the distribution of hCYP1A2 and hCYP3A4 in the chimeric mouse liver lobules following administration of 3-MC (20 mg/kg) and rifampicin (50 mg/kg) once daily for four days, immunohistochemistry was conducted using liver specimens from the chimeric mice. These liver specimens were derived as reported previously [2]. Chimeric mice with nine-month-old male Caucasian (9MM) and 12-year-old male Caucasian (12YM) donor cells were used to evaluate the distribution of hCYP1A2 and hCYP3A4, respectively. Livers were removed at 24 h after the last administration of inducers and then processed for double immunohistochemistry. Livers from non-treated mice were used as controls (Table 7). Cryosections (5 μm thick) were incubated with anti-human-specific cytokeratin 8 and 18 (hCK8/18) antibodies (ICN Pharmaceuticals, Inc, Aurora, OH, USA), and rabbit anti-human CYP1A2 or CYP3A4 polyclonal antibodies (Affiniti Research Products, Ltd., Exeter, UK). Immunoreactions for hCK8/18 and hCYP1A2 or hCYP3A4 were visualized with FITC-conjugated anti-mouse IgG (Fc) (Pierce Biotechnology, Inc. Rockford, IL, USA) and Texas Red-conjugated anti-rabbit IgG (H + L) (Vector Laboratories, Inc. Burlingame, CA, USA), respectively. These antibodies were confirmed to have human-specific reactivity (data not shown).

### 3.6. Preparation of Fresh Human Hepatocytes from Chimeric Mice

Human hepatocytes were isolated from chimeric mice by a two-step collagenase perfusion method as reported previously [10]. After completion of the perfusion, liver cells were disaggregated in CMF-HBSS containing 10% bovine albumin, 10 mM Hepes, and 10 mg/mL gentamycin. The disaggregated cells were centrifuged three times (50 ×*g*, 2 min). The pellet was suspended in medium consisting of Dulbecco’s modified Eagle’s medium (DMEM) with 10% fetal bovine serum (FBS), 20 mM Hepes, 44 mM NaHCO_3_, and antibiotics (100 IU/mL penicillin G and 100 μg/mL streptomycin) (DMEM10). Cell number and viability were estimated by the trypan blue exclusion test. The cell number (yield) of isolated viable hepatocytes was 4.5 × 10^7^–10.9 × 10^7^ cells/mouse, and the viability was 65.3%–80.3% (Table 6).

### 3.7. *In Vitro* CYP Induction Study

The isolated human hepatocytes (PXB-cells) in DMEM10 medium were immediately inoculated on uncoated 24-well plates (Corning Life Science, Tewksburry, MA, USA) for the monolayer culture and Matrigel-coated 24-well plates (BD Biosciences, San Jose, CA, USA) for the spheroid culture at a cell density of 5 × 10^4^ cells/cm^2^ [22]. At 5 h post inoculation, the medium was changed to remove dead cells. Then, 19 h later, the medium was changed to serum-free medium (HHM; Toyobo, Osaka, Japan) and the cells were cultured for 24 h. The induction began after the completion of the formation of both the monolayer culture on uncoated 24-well plates and the spheroid culture on Matrigel-coated 24-well plates. The medium was changed to HHM containing the solvent control, 10, 50, or 250 ng/mL 3-MC, or 5 or 25 μg/mL rifampicin. At 24 h (72 h post inoculation) and 48 h post induction (96 h post inoculation), cells were harvested in RLT buffer (Qiagen K.K., Tokyo, Japan) to isolate total RNA for determining hCYP1A1, hCYP1A2, and hCYP3A4 mRNA expression levels by real-time qRT-PCR and stored at −80 °C until use.

### 3.8. Determination of mRNA Expression Levels by Real-Time qRT-PCR

Total RNA was isolated from the harvested hepatocytes or chimeric mouse livers and then treated with DNase (Qiagen, Tokyo, Japan), purified using the RNase-Free DNase Set (Qiagen, Tokyo, Japan) and the RNeasy Mini Kit (Qiagen, Tokyo, Japan). mRNA expression levels of h*CYP1A1*, h*CYP1A2*, and h*CYP3A4* were quantified by real-time qRT-PCR. cDNA was synthesized using 1 μg RNA, PowerScript reverse transcriptase (Clontech, Mountain View, CA, USA), and random primers (Life Technologies Corp., Carlsbad, CA, USA) according to the manufacturer’s protocol, and was subjected to real-time qRT-PCR. Genes were amplified with a set of gene-specific primers (Table 8) and the SYBR Green PCR mix in a PRISM 7700 Sequence Detector (Life Technologies Corporation, Carlsbad, CA, USA). We confirmed that these primers were capable of amplifying human, but not mouse genes. Levels of PCR products were monitored continuously during amplification by measuring the increases in intensity of SYBR Green 1 that bound to the double-stranded DNA. The PCR conditions consisted of an initial denaturation step at 95 °C for 10 min, followed by 40 cycles of 95 °C for 15 s, and 60 °C for 1 min. Results were calculated by the comparative threshold cycle (*C_t_*) method, as described previously [23]. To normalize the human mRNA expression levels in total RNA samples, the expression of human CYP mRNA was divided by the value for h*GAPDH* mRNA individually, because the extracted total RNA sample contained varying amounts of total RNA derived from mouse tissue.

### 3.9. Western Blotting

Microsomal fractions were isolated from chimeric mice livers [24], aliquots of which (25 μg of protein) were loaded on 10% SDS-polyacrylamide gels, electrophoresed, and transferred to nitrocellulose membranes. The membranes were incubated with antibodies against hCYP1A2 (Sekisui Medical Co., Ltd., Tokyo, Japan) and hCYP3A4 (Sekisui Medical Co., Ltd., Tokyo, Japan) and visualized using the ECL Western Blotting Detection System (Amersham Biosciences Corp., Piscataway, NJ, USA). The intensity of the detected color on the nitrocellulose membrane was measured using the ImageJ software (ver. 1.43; NIH, Bethesda, MD, USA).

### 3.10. Statistical Analyses

Statistical differences were evaluated with a homogeneity of variance test (Bartlett’s test), followed by Dunnett’s and Steel’s tests.

## 4. Conclusions

We report here for the first time, CYP induction levels determined for inducers *in vivo* and *in vitro* using cells from the same donor. The comparison between *in vivo* and *in vitro* data using the chimeric mice can provide valuable information for predicting the CYP-inducing abilities of new drug candidates, both quantitatively and qualitatively. The present study demonstrated that a chimeric mouse with a humanized liver is a unique tool for evaluating enzyme induction, and we propose using the chimeric mice in both *in vivo* and *in vitro* enzyme induction studies for advancing predictions of enzyme-inducing effects in humans.

## Figures and Tables

**Table 1.** PK profile in *in vivo* study.

Inducer	Dose (mg/kg)	Day	*AUC* (1) (Ratio to Day 0) (ng/mL·h)	*T*_1/2_ (h)	*C*_max_ (1) (ng/mL)	*T*_max_ (h)
3-MC	2	0	44.2 ± 0.0	2.1	7.4 ± 0.0	2.0
	-	3	0.0 ± 0.0 (0.0)	-	-	-
	20	0	94.4 ± 20.8	8.6	12.2 ± 4.3	2.0
	-	3	35.4 ± 21.3 ** (0.4)	9.1	2.4 ± 0.3	2.0

Rifampicn	0.1	0	236.8 ± 76.6	3.1	45.0 ± 19.2	0.5
	-	3	154.9 ± 47.6 (0.7)	3.5	24.1 ± 8.7	0.5
	10	0	40,186.5 ± 9,088.3	3.0	6,926.0 ± 4,653.4	0.5
	-	3	22,125.3 ± 6,222.4 (0.6)	2.0	5,353.3 ± 1,122.5	1.0

(1) mean ± S.D.;

** *p* < 0.01.

**Table 2.** mRNA expression levels in *in vivo* study.

Group	Dose (mg/kg)	h*CYP1A1*/h*GAPDH* (1) (Ratio to the Control)	h*CYP1A2*/h*GAPDH* (1) (Ratio to the Control)	h*CYP3A4*/h*GAPDH* (1) (Ratio to the Control)
Corn oil	-	0.112 ± 0.041 (1.0)	2.576 ± 1.081 (1.0)	2.471 ± 0.804 (1.0)

3-MC	2	0.156 ± 0.037 (1.4)	3.091 ± 0.850 (1.2)	-
	20	0.812 ± 0.217 * (7.3)	5.666 ± 3.283 (2.2)	-

Rifampicin	0.1	-	-	2.718 ± 0.375 (1.1)
	10	-	-	12.519 ± 6.296 * (5.1)

(1) mean ± S.D.;

* *p* < 0.05.

**Table 3.** Protein expression levels in *in vivo* study.

Group	Dose (mg/kg)	hCYP1A2 (1) (Ratio to the Control)	hCYP3A4 (1) (Ratio to the Control)
Corn oil	-	6,906.7 ± 488.0	2169.0 ± 527.7

3-MC	2	6,220.7 ± 1,082.8 (0.9)	-
	20	10,389.0 ± 1,943.0 * (1.5)	-

Rifampicin	0.1	-	1,798.3 ± 372.1 (0.8)
	10	-	4,796.3 ± 1,198.0 * (2.2)

(1) mean ± S.D.;

* *p* < 0.05.

**Table 4.** mRNA expression levels in *in vitro* study.

Item	Group	Monolayer Culture		Spheroid Culture	
	
		24 h (1) (Ratio to the Control)	48 h (1) (Ratio to the Control)	24 h (1) (Ratio to the Control)	48 h (1) (Ratio to the Control)
h*CYP1A1*/h*GAPDH*	Control	0.022 ± 0.033 (1.0)	0.033 ± 0.054 (1.0)	0.018 ± 0.021 (1.0)	0.013 ± 0.007 (1.0)
	3-MC 10 ng/mL	0.053 ± 0.060 (2.4)	0.038 ± 0.056 (1.2)	0.047 ± 0.035 (2.6)	0.023 ± 0.018 (1.8)
	3-MC 50 ng/mL	0.112 ± 0.101 (5.1)	0.053 ±0.053 (1.6)	0.120 ± 0.073 (6.6)	0.053 ± 0.028 (4.2)
	3-MC 250 ng/mL	0.314 ± 0.170 * (14.3)	0.158 ± 0.086 (4.9)	0.315 ± 0.175 ** (17.3)	0.186 ± 0.087 (14.9)

h*CYP1A2*/h*GAPDH*	Control	0.007 ± 0.006 (1.0)	0.003 ± 0.004 (1.0)	0.139 ± 0.091 (1.0)	0.168 ± 0.062 (1.0)
	3-MC 10 ng/mL	0.015 ± 0.011 (2.3)	0.004 ± 0.004 (1.4)	0.215 ± 0.106 (1.6)	0.228 ± 0.056 (1.4)
	3-MC 50 ng/mL	0.017 ± 0.008 (2.5)	0.006 ± 0.004 (1.8)	0.392 ± 0.069 ** (2.8)	0.423 ± 0.117 * (2.5)
	3-MC 250 ng/mL	0.033 ± 0.012 * (4.9)	0.015 ± 0.004 ** (4.9)	0.713 ± 0.023 ** (5.1)	0.967 ± 0.111 ** (5.8)

h*CYP3A4*/h*GAPDH*	Control	0.019 ± 0.004 (1.0)	0.004 ± 0.000 (1.0)	0.278 ± 0.240 (1.0)	0.116 ± 0.070 (1.0)
	Rifampicin 5 μg/mL	0.075 ± 0.044 * (4.0)	0.040 ± 0.020 * (10.9)	4.138 ± 0.405 ** (14.9)	3.421 ± 1.601 * (29.4)
	Rifampicin 25 μg/mL	0.187 ± 0.061 * (9.8)	0.055 ± 0.025 * (15.1)	4.952 ± 1.294 ** (17.8)	2.770 ± 0.907 * (23.8)

(1) mean ± S.D.;

* *p* < 0.05;

** *p* < 0.01.

**Table 5.** Chimeric mice used in the *in vivo* study.

Donor Cells	Group	Dose (mg/kg)	No. of Animals	Age (weeks)	hAl in Blood (1) (Min–Max) (mg/mL)	Body Weight (1) (Min–Max) (g)	RI *,(1) (%)
10YF	Corn oil	-	3	12–13	8.2 ± 2.4 (6.7–10.9)	18.7 ± 1.2 (17.4–19.8)	79 ± 8 (74–89)
	3-MC	2	3	12–14	8.3 ± 2.1 (6.7–10.6)	19.7 ± 1.9 (17.5–21.3)	80 ± 7 (74–88)
		20	3	13–14	8.2 ± 2.1 (6.5–10.5)	17.8 ± 3.7 (14.2–21.5)	80 ± 8 (73–88)
	Rifampicin	0.1	3	12–13	7.5 ± 1.0 (6.4–8.4)	19.6 ± 3.7 (15.5–22.8)	77 ± 5 (72–81)
		10	3	13–14	7.6 ± 1.1 (6.3–8.5)	19.6 ± 2.4 (17.5–22.2)	77 ± 5 (72–81)

(1) mean ± S.D.;

* expected RI. RI calculated by the blood hAlb levels using the formula of the correlation curve *y* = 30.4 ln (*x*) + 16.0 (*r*^2^ = 0.88) in which *x* and *y* represent *r*^2^ and hAlb level, respectively.

**Table 6.** Chimeric mice used in the *in vitro* study.

Donor Cells	hAl in Blood (mg/mL)	RI * (%)	Cell Yield (×10^7^ Cells)	Viability (%)
10YF	14.8	98	4.5	75.1
	11.0	89	8.1	65.3
	8.1	80	10.9	80.3
	6.2	72	10.2	65.8

* expected RI. RI calculated by the blood hAlb levels using the formula of the correlation curve *y* = 30.4 ln (*x*) + 16.0 (*r*^2^ = 0.88) in which *x* and *y* represent *r*^2^ and hAlb level, respectively.

**Table 7.** Donor information and details of treatment of the liver samples for immunohistochemistry.

Donor Cells	Group	Dose (mg/kg)	No. of Animals	Age (weeks)	hAl in Blood (mg/mL)	Body Weight (g)	RI * (%)
9MM	Control	-	6	12–14	1.4–5.7	9.0–21.8	1–57
	3-MC	20	3	12–13	1.4–10.7	12.9–13.8	6–76

12YM	Control	-	6	10–14	0.03–5.1	5.1–22.8	41–89
	Rifampicin	50	3	16–18	0.07–4.6	13.5–16.5	45–49

* expected RI. RI calculated by the blood hAlb levels using the formula of the correlation curve *y* = 13.3 ln (*x*) + 48.6 (*r*^2^ = 0.76) for 9MM and in *y* = 19.3 ln (*x*) − 227.0 (*r*^2^ = 0.63) for 12YM in which *x* and *y* represent *r*^2^ and hAlb level, respectively.

**Table 8.** Primers used in this study.

Target Gene	Primer	Sequence
*hCYP1A1*	Forward	TCAACCATGACCAGAAGCTA
	Reverse	AAGATAATCACCTTCTCACTTAACAC

*hCYP1A2*	Forward	GCTTCTACATCCCCAAGAAAT
	Reverse	ACCACTTGGCCAGGACT

*hCYP3A4*	Forward	ACTGCCTTTTTTGGGAAATA
	Reverse	GGCTGTTGACCATCATAAAAG

*hGAPDH*	Forward	GGAGTCAACGGATTTGGT
	Reverse	AAGATGGTGATGGGATTTCCA
